# Successful treatment of pancreatic schwannoma by enucleation: A case report

**DOI:** 10.1097/MD.0000000000028874

**Published:** 2022-03-04

**Authors:** Shao-Yan Xu, Bo Zhou, Shu-Mei Wei, Ya-Nan Zhao, Sheng Yan

**Affiliations:** a Division of Hepatobiliary and Pancreatic Surgery, Department of Surgery, Second Affiliated Hospital, School of Medicine, Zhejiang University, Zhejiang Province, Hangzhou, China; b Key Laboratory of Precision Diagnosis and Treatment for Hepatobiliary and Pancreatic Tumor of Zhejiang Province, Hangzhou, Zhejiang Province, China; c Department of Pathology, Second Affiliated Hospital, School of Medicine, Zhejiang University, Zhejiang Province, Hangzhou, China; d Department of Ultrasound, Second Affiliated Hospital, School of Medicine, Zhejiang University, Zhejiang Province, Hangzhou, China.

**Keywords:** case report, enucleation, pancreas, S-100, schwannoma

## Abstract

**Rationale:**

Pancreatic schwannomas are extremely rare and are difficult to diagnose preoperatively. Over the past 50 years, only 96 cases of pancreatic schwannoma have been reported in English literature. Herein, we report a case of pancreatic schwannoma treated with enucleation.

**Patient concerns:**

A 66-year-old woman visited a local hospital due to ventosities. Ultrasonography and computed tomography revealed a pancreatic mass. She visited our hospital for further diagnosis and treatment.

**Diagnosis and interventions:**

Magnetic resonance imaging revealed a tumor in the pancreatic body, and a solid pseudopapillary tumor was considered preoperatively. During the surgery, a pancreatic mass was found growing in the pancreatic body and tail. A successful tumor enucleation was performed. The mass was 7 × 6 × 3 cm in size with a thin capsule. Pathological examination revealed that the tumor was mainly composed of spindle-shaped cells with a palisading arrangement and no atypia. Both hypercellular and hypocellular areas were visible. Immunohistochemical staining showed that protein S-100 was strongly positive. The tumor was diagnosed as a benign schwannoma originating from the pancreatic body and tail.

**Outcomes:**

Postoperatively, the patient showed good recovery. During the 24-month follow-up period, the patient remained well and free of complications.

**Lessons:**

Pancreatic schwannomas are extremely rare and difficult to diagnose using imaging examinations. Enucleation is a safe and efficacious treatment for exophytic pancreatic schwannomas.

## 1 Instruction

Schwannomas are mesenchymal tumors originating from Schwann cells that wrap around each axon to form a myelin sheath in myelinated nerve fibers.^[1]^ Schwannomas are generally encapsulated tumors, the majority of which are benign. Malignant cases are rarely reported and often have a cystic formation and/or large tumor size, occurring in approximately 5% of patients with von Recklinghausen's disease.^[2]^ Schwannomas often contain a solid component with areas of degenerative change, such as cysts, calcification, hemorrhage, and hyalinization. Most schwannomas show either monosomy 22 or loss of 22q material. The pathogenesis of this tumor remains unclear.^[2]^ Patients between 20 and 50 years of age are most frequently reported to have schwannomas, with no obvious gender differences. Surgery is usually performed to treat these tumors and patients have a good prognosis.^[1]^ Tumors can involve almost every part of the human body and are often detected in the head and neck, extremities, mediastinum, and retroperitoneum.^[3]^ However, pancreatic schwannomas are rare. To the best of our knowledge, only 96 cases of pancreatic schwannomas have been reported in the English literature over the past 50 years.^[4–83]^ Most patients are asymptomatic, and the tumor is incidentally found. Here, we present a case of pancreatic schwannoma in a 66-year-old woman who was treated with enucleation and had a good prognosis.

## 2 Case presentation

A 66-year-old woman visited a local hospital due to ventosities. A pancreatic mass was identified using ultrasonography (US) and computed tomography (CT) at a local hospital. The patient was referred to our hospital for further diagnosis and treatment. She had a history of hypertension for 10 years and had undergone cataract surgery in her left eye 3 years ago. Partial thyroidectomy was performed 2 years ago because of nodular goiter. One year prior, she had undergone colon polyp excision under colonoscopy. The patient's vital signs were stable. The abdomen was soft and non-distended, without evidence of a palpable mass. Levels of the tumor markers alpha-fetoprotein, cancer antigen-199, cancer antigen-125, and carcinoembryonic antigen were within the normal range. Blood tests, fecal examinations, and coagulation function tests were normal.

**Figure 1:**
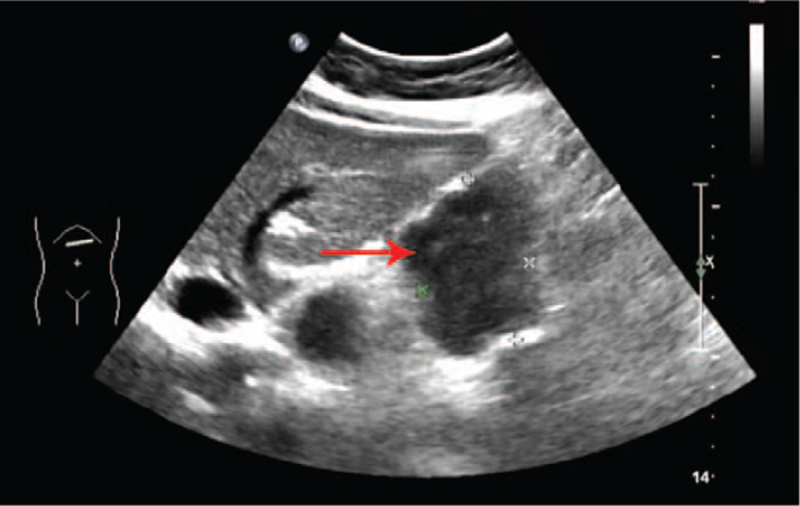
Ultrasound findings. Ultrasound revealed a 5.6 × 3.6 cm, inhomogeneous hypoechoic well-defined lesion (red arrow) in the pancreatic tail.

**Figure 2:**
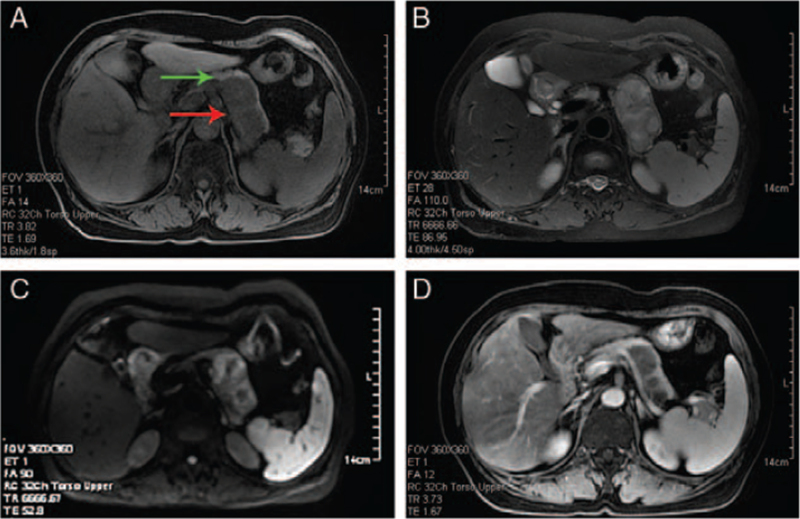
Magnetic resonance imaging (MRI) findings. A: The mass (red arrow) in the pancreas (green arrow) appeared hypointense on T1 weighted images. B: The mass in the pancreatic body appeared inhomogeneous hyperintense on T2 weighted images. C: The tumor appeared inhomogeneous hyperintense on diffusion-weighted imaging. D: On the enhanced MRI, the tumor appeared heterogeneously enhanced.

On US, an inhomogeneous hypoechoic mass 5.5 × 3.6 cm in size was detected in the pancreatic tail (Fig. 1) and the boundary was discernable. Magnetic resonance imaging (MRI) revealed that the mass in the pancreatic tail appeared hypointense on T1 weighted images (Fig. 2A). The mass in the pancreatic tail appeared inhomogeneous hyperintense on T2 weighted images (Fig. 2B). It also appeared inhomogeneous hyperintense on diffusion-weighted imaging (Fig. 2C). On enhanced MRI, the tumor appeared heterogeneously enhanced (Fig. 2D). Based on these results, a solid pancreatic pseudopapillary tumor was preliminarily considered.

After obtaining sufficient preparation and consent from the patient and her family members, laparotomy was performed. An 8 × 5 cm mass surrounded by a thin fibrous capsule was found in the pancreatic body and tail with exophytic growth. Tumor enucleation was performed. Intraoperative frozen pathology revealed a spindle cell tumor in the pancreatic tail, which was considered to be mesenchymal in origin and possibly benign.

**Figure 3:**
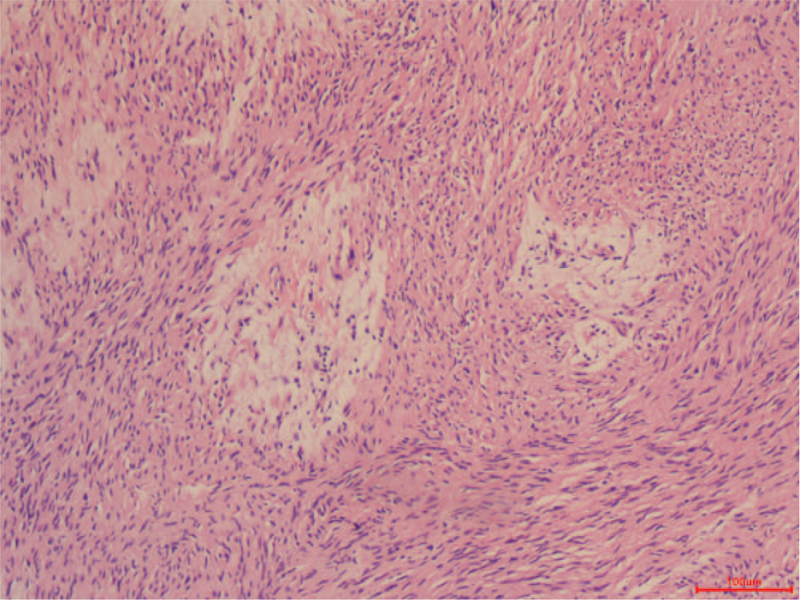
Microscopic examination. The tumor was surrounded by a thin capsule and was mainly composed of spindle-shaped cells with a palisading arrangement and no atypia. Both hypercellular and hypocellular areas were observed (HE, 100 ×). HE: Hematoxylin and eosin.

**Figure 4:**
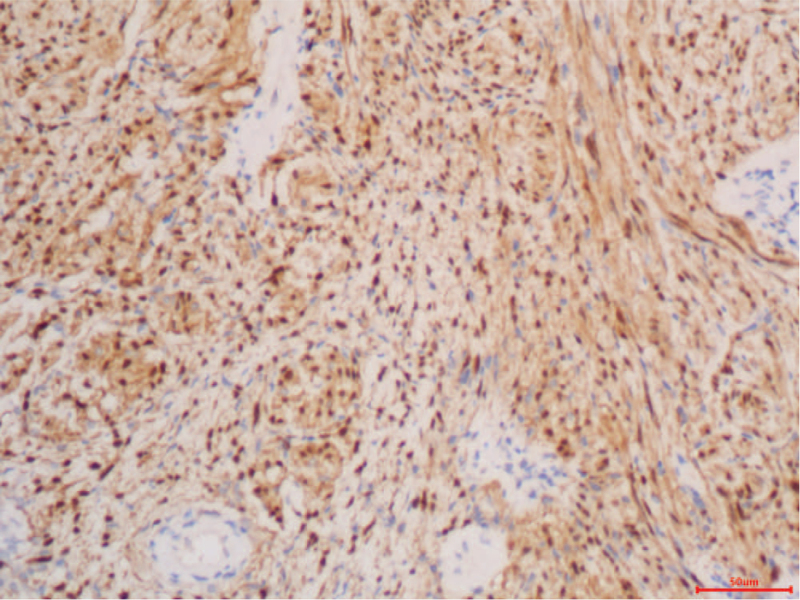
Immunohistochemical staining. The tumor revealed strong positive staining for S-100 (HE, 200 ×). HE: Hematoxylin and eosin.

Macroscopically, the mass in the pancreatic body and tail measured 7 × 6 × 3 cm. Microscopically, the tumor was surrounded by a thin capsule and was mainly composed of spindle-shaped cells with a palisading arrangement and no atypia. Both hypercellular and hypocellular areas were observed (Fig. 3). Immunohistochemistry revealed strong positive staining for protein S-100 (Fig. 4) and negative results for smooth muscle actin (SMA), CD34, and CD117. In this case, the final diagnosis was pancreatic schwannoma. Postoperatively, the patient had a grade A pancreatic fistula, but she recovered well and was discharged from the hospital 6 days later. During the 24-month follow-up period, the patient remained healthy without any complications.

## 3 Discussion

Schwannomas are neoplasms that originate from Schwann cells in nerve sheaths.^[84]^ More than 90% of schwannomas are benign, are usually encapsulated, and grow slowly. Approximately 10% of cases are associated with genetic disorders such as neurofibromatosis type 2, multiple meningiomas, and schwannomatosis. Few cases are associated with neurofibromatosis type 1.^[85]^ Schwannomas usually occur in adults with a slightly higher incidence in women than in men. The head, neck, and extremities are the most commonly sites involved.^[86]^ Schwannomas in the abdominal cavity, such as the retroperitoneum (6% of primary retroperitoneal tumors)^[87]^ and stomach,^[88]^ have also been reported. However, schwannomas of the pancreas are rare and arise from either sympathetic or parasympathetic nerve fibers coursing through the pancreas. Over the past 50 years, only 96 pancreatic schwannomas have been reported in English literature. Of the 97 patients included in this study, the male/female ratio was 42.71%. The patients’ ages ranged from 20 to 87 years (mean age, 55.51 years). The pancreatic head was the most frequently involved location (34.02%). Nearly half of the patients were asymptomatic (46.88%).

Precise preoperative diagnosis of pancreatic schwannomas is difficult because the clinical symptoms and radiological characteristics of schwannomas are nonspecific. Definitive diagnosis is achieved only through the combined results of histopathological and immunohistochemical examinations of the surgical specimens. Macroscopically, pancreatic schwannomas are usually well-circumscribed, encapsulated, homogeneous, yellow-tan masses. Secondary degenerative changes, such as cyst formation, hemorrhage, hyalinization, and calcification, can occur in more than half of pancreatic schwannomas.^[40]^ Microscopically, a typical schwannoma has two main microscopic patterns of growth: Antoni A (hypercellular component) and Antoni B (hypocellular component).^[22]^ The hypercellular area consisted of closely packed spindle cells with occasional nuclear palisading. The hypocellular area is composed of loosely arranged tumor cells and abundant myxoid stroma.^[28]^ Pancreatic schwannomas demonstrate strong positive immunohistochemical staining for protein S-100 and negative staining for SMA, CD34, CD117, desmin, and smooth muscle myosin.^[38,89]^ Most pancreatic schwannomas are benign and only five malignant cases have been reported so far.^[44,79,81–83]^

A variety of diagnostic imaging modalities, such as US, CT, and MRI, can be used to establish a probable diagnosis. On US, a pancreatic schwannoma usually appears as a well-defined hypodense lesion and shows no echoic enhancement on color Doppler imaging. On unenhanced CT, schwannomas are usually well-defined hypodense lesions with encapsulation and can show cystic degeneration. In areas of Antoni A, they appear heterogeneous, solid, and hypodense owing to their compact cellular organization and high lipid content in the tumor. The Antoni B areas of schwannomas show low density due to loose stroma and low cellularity.^[32]^ On contrast-enhanced CT, Antoni A areas are usually enhanced due to well-developed reticular vascular components, whereas Antoni B areas are unenhanced due to less vascularity.^[29]^ On MRI, a typical schwannoma appears hypointense on T1-weighted images and inhomogeneous hyperintense on T2-weighted images with encapsulation.^[7]^ Following gadopentetic acid administration, most pancreatic schwannomas can be gradually enhanced on T1-weighted images.^[7]^ On 18F-fluorodeoxyglucose positron emission tomography (FDG-PET), elevated FDG activity in schwannomas has been reported,^[90]^ even though they are usually benign. To date, all 5 cases of pancreatic schwannoma that underwent PET/CT had increased FDG uptake.^[8,17,23,29]^ Recently, an increasing number of cases have undergone endoscopic ultrasound fine needle aspiration (EUS-FNA), which has greatly contributed to precise preoperative diagnosis. Of the 12 patients with pancreatic schwannoma who underwent EUS-FNA, 9 were accurately diagnosed. Observation was conducted in seven patients, and surgery was avoided.^[14]^ Thus, endoscopic ultrasound-guided fine needle aspiration is necessary for preoperative diagnosis of pancreatic schwannomas.

Surgery is the curative treatment for pancreatic schwannomas, and most cases are treated via laparotomy. Because the tumor can be located in different sections of the pancreas, surgical approaches may vary, such as pancreaticoduodenectomy, distal pancreatectomy, and central pancreatectomy. Enucleation was reported in 10 patients. In the present case, we performed enucleation of an exophytic mass in the pancreatic body and tail. If a precise preoperative diagnosis can be made and the patient is asymptomatic, surveillance may be a good choice. After complete tumor excision, patients with pancreatic schwannomas generally have a good prognosis.

## 4 Conclusion

Pancreatic schwannomas are rare. To the best of our knowledge, only 96 cases of pancreatic schwannoma have been reported in the English literature over the past 50 years. Precise preoperative diagnosis is challenging despite the use of multiple imaging modalities. Surgery is the most common treatment for pancreatic schwannomas. As tumors can be located in different parts of the pancreas, the surgical methods vary accordingly. Recently, an increasing number of cases have achieved an accurate preoperative diagnosis following EUS-FNA, and surveillance is also a good option. Patients with pancreatic schwannomas usually have a good prognosis after resection.

## Author contributions

**Conceptualization:** Shaoyan Xu, Sheng Yan.

**Funding acquisition:** Sheng Yan.

**Resources:** Shu-Mei Wei, Ya-Nan Zhao.

**Writing – original draft:** Shaoyan Xu.

**Writing – review & editing:** Bo Zhou, Sheng Yan.
